# Fast Track Surgery as the Latest Multimodal Strategy of Enhanced Recovery after Urethroplasty

**DOI:** 10.1155/2023/2205306

**Published:** 2023-05-11

**Authors:** Vladimir Beloborodov, Vladimir Vorobev, Temirlan Hovalyg, Igor Seminskiy, Svetlana Sokolova, Ekaterina Lapteva, Aleksandr Mankov

**Affiliations:** ^1^Department of General Surgery, Irkutsk State Medical University, Irkutsk, Russia; ^2^Department of Phatology, Irkutsk State Medical University, Irkutsk, Russia; ^3^Department of Geriatrics, Propaedeutics and Management in Nursing, North-Western State Medical University Named after I.I. Mechnikov, Saint Petersburg, Russia; ^4^Department of Anesthesiology-Resuscitation, Irkutsk State Medical University, Irkutsk, Russia

## Abstract

Fast track surgery (FTS), as well as ERAS (enhanced recovery after surgery/rapid recovery programs), is the latest multimodal treatment strategy, designed to reduce the disability period and improve the medical care quality. The study aims to analyze the enhanced recovery protocol effectiveness in a comparative study of elective urethral stricture surgery. A prospective study included 54 patients with an established diagnosis of urethral stricture in 2019-2020 in the urological hospital of the Irkutsk City Clinical Hospital No. 1. All 54 patients have completed the study. There were two groups of patients FTS-group (group II, *n* = 25) and standard group (group I, *n* = 29). In terms of preoperative parameters, the comparison groups have statistical homogeneity. The comparative intergroup efficacy analysis of the treatment based on the criteria established in the study demonstrated good treatment results for 5 (17.2%) patients of group I and 20 (80%) patients of group II (*p*=0.004). The overall efficacy of urethroplasty surgeries, regardless of the treatment protocol, was comparable (86.2% vs 92%; *p*=0.870), as well as the likelihood of relapse within two years (*p*=0.512). The predictors of recurrence were technical complications and urethral suture failure (OR 4.36; 95% CI 1.6; 7.11; *p*=0.002). The FTS protocol reduced the treatment period (*p* < 0.001) and decreased the severity of postoperative pain (*p* < 0.001). The use of the “fast track surgery” protocol in urethroplasty with generally similar treatment results makes it possible to achieve a better functional and objective condition of patients in the postoperative period due to less pain, shorter catheterization, and hospitalization.

## 1. Introduction

Fast track surgery (FTS), as well as ERAS (enhanced recovery after surgery/rapid recovery programs), is the latest multimodal treatment strategy designed to reduce disability periods and improve the quality of medical care. This program includes the preoperative stage preparation, minimally invasive surgical techniques, and active management of the post-operative stage to reduce the treatment period, rehabilitation time and to provide the fastest possible return of patients to normal life. In this article, the FTS and ERAS (enhanced recovery after surgery) protocols are equivalent, pursuing the same goal.

The main objectives of the FTS are multidisciplinary interaction at all stages of examination and treatment [1], assessment of the applied protocol for compliance with the FTS principles [2], careful selection of patients for the program for various reasons, including religious reasons, which may hinder participation [3]. There is an assessment of the risks of using the protocol, which may increase the likelihood of postoperative complications or negative outcomes due to the impossibility of adhering to the protocol (due to lifestyle or concomitant diseases) [4]. The program also includes a revision of the concept of preoperative and postoperative food and liquid intake and bowel preparation [5–7]. As well as changing opiate and opiate-like analgesics to nonsteroidal drugs and others [8]; implementation of multimodal anesthesia allowing minimizing operational and post-operative stress in combination with early mobilization [9], intraoperative normothermia maintenance [10], control of fluid [11], post-operative pain [9, 12], and nausea [13].

There are critically little works on FTS in urethroplasty. Selected studies and inventions evaluated applicability in the developed protocol. This made it possible to apply the concept of using fibrin glue [14] and platelet-rich plasma (PRP) [15]. The submucosal PRP injection for internal optical urethrotomy significantly reduces the risk of recurrence (9.09% vs 26.82%, p 0.032) in the long-term postoperative period [16].

Currently, the number and quality of randomized researches devoted to the fast track protocols in reconstructive urology are extremely small. At the same time, the problem of preoperative preparation, optimization of surgical techniques, and acceleration of postoperative rehabilitation remain relevant, since there have been no significant changes in approaches to perioperative management of patients over the past ten years.

The scientific novelty of the study is due to several factors. A set of fast track and ERAS measures will be evaluated for patients with planned urethroplasty, aimed at improving all aspects of treatment (patient and medical staff satisfaction with treatment, increased treatment effectiveness, improved financial efficiency of treatment, and accelerated rehabilitation of patients after treatment). For the first time, there will be a comparative analysis of the immediate and long-term results of the application of the developed principles and the standard approach. The study will present the evaluation of postoperative complications associated with various methods of treatment; the results of the application of the developed and traditional approaches; and predictors of complications in the long-term postoperative period.

Research objectives: to develop and adapt the FTS protocol for patients with planned urethroplasty; to increase the overall and financial efficiency of surgical treatment; to assess the impact of the compared approaches on the condition of patients and their satisfaction with the treatment; to analyze the immediate and long-term results; and to develop recommendations for the developed protocol of FTS urethroplasty.

The study aims to analyze the effectiveness of Enhanced Recovery Protocol in a comparative study for elective urethral stricture surgery.

## 2. Materials and Methods

### 2.1. Research Design

The local ethics committee of the Irkutsk State Medical University (ISMU) and the Irkutsk City Clinical Hospital No. 1 approved the clinical trial. There was a prospective, blind, randomized, and single-center study in the urological hospital of the Irkutsk City Clinical Hospital No. 1.

The clinical part of the study includes an analysis of the examination and treatment results of patients who underwent surgical interventions for urethral stricture from January 2019 to August 2020. Surgeries were performed using anastomotic and augmentation/substitution methods.

The inclusion criteria were as follows:

- The patient is planned to have surgery of the urethra for urethral stricture or distraction defect of the urethra
- Indications for surgery meet the criteria of the approved protocol
- The operation is planned using one of the methods approved in the study
- Patients are over 18 years old
- The patient signed a voluntary informed consent to participate in the study
- The patient is deliberately planned to follow the treatment protocol (FTS or standard), determined by randomization until the day of surgery

Noninclusion criteria were as follows:

Lack of medical indications;

- The patient did not sign the voluntary informed consent form to participate in the study
- The concomitant diseases that significantly affect the patient's objective status (decompensated diabetes mellitus, heart failure, gross neurological deficits, etc.)
- Failure to comply with the FTS protocol

Exclusion criteria we as follows:

- The patient refused to participate at any stage of the study
- For any reason, there was a deviation from the research protocol
- The patient did not have surgery for any reason or underwent another surgery that does not meet the group's criteria

The null hypothesis of the study was that there were no between-group differences on the primary point. If the null hypothesis was rejected, the alternative hypothesis was that there was an intergroup difference at the primary endpoint.

Taking the results of earlier studies on similar subjects, it was calculated that 13–15 (*t*-test, ES = −1.136) patients in each comparison group would be sufficient to reproduce differences in success and postoperative status with odds of type 1 and type 2 error of 0.05 and 0.2, respectively. Study power is >0.8. To compensate for incomplete observations, the estimated sample size was increased by 10%.

Thus, the required total sample size (two patient comparison groups) should be at least 33 patients.

**Figure 1:**
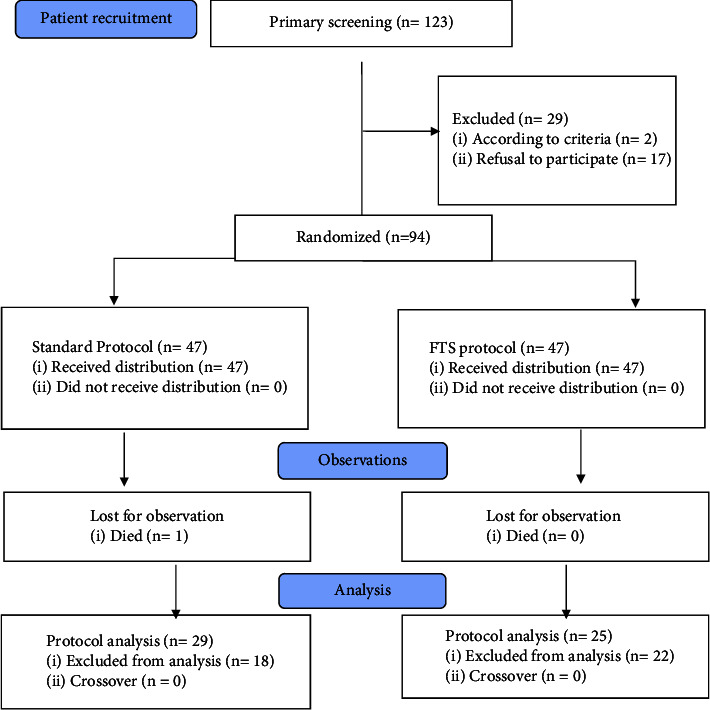
CONSORT research diagram.

The recruitment of patients who met the inclusion criteria was carried out prospectively using the continuous sampling method until the desired sample size was reached. During the indicated period, 123 patients had the diagnosed stricture disease of the urethra. Only 94 patients met the study inclusion criteria. All included patients were randomized (simple randomization method) into two groups based on the approved study protocol. The first group followed the FTS treatment protocol (approved by the ethical committee of the ISMU); the second group followed the standard treatment protocol (Figure 1).

Thus, the final clinical analysis included 54 patients (per-protocol) who met all the study criteria. They formed two groups. The group of patients with the standard treatment protocol (*n* = 29, the group I), and the group with the FTS protocol (*n* = 25, group II).

### 2.2. Checkpoints

The study has checkpoints.

The primary “hard” checkpoints were the absence of recurrence of urethral stricture in the late postoperative period (but not earlier than three months); and detected relapse at any stage of postoperative follow-up.

Secondary “soft” checkpoints of clinical efficacy were the data of the subsequent postoperative examination: maximum urine flow rate of more than 12 ml/sec, residual urine volume less than 50 ml, indicators of the IIEF-5, IPSS, QoL scales, and no signs of recurrence according to urethrography data (diameter of the urethral lumen more than 5 mm).

### 2.3. Study Groups Comparison

Table 1 presents the comparative data on the initial parameters of patients in the study groups.

Table 2 presents comparative data of the results of an objective examination and the state of the functional status of the comparison groups.

### 2.4. Diagnostic Methods

The examination included anamnestic (to establish the duration of the disease, concomitant diseases, etc.), clinical, biochemical, ultrasound, tomographic, radiological, and endoscopic research methods.

To clarify the nature and degree of pathological changes in the urethra there was urethrocystography (X-ray or MSCT) and urethrocystoscopy. An ultrasound helped to assess the volume of residual urine and the volume of the prostate, uroflowmetry data showed the maximum urine flow rate (*Q*_max_). When visiting a doctor, patients had to assess their subjective state and functional status according to the recommended scales for assessing lower urinary tract symptoms (LUTS/IPSS), quality of life (QoL), erectile function (IIEF-5), and other parameters. The visual analog scale of pain (VAS) helped to assess the severity of postoperative pain syndrome. These assessments of the subjective state were as follows: 0-1 points-no pain; 2-3 points-slight pain; 4-5 points-moderate intermittent pain; 6-7 points-moderate persistent pain; 8-9 points-severe pain; more than 10 points-unbearable pain.

Before removal of the urethral catheter, there was a pericatheter urethrography to assess a possible defect in the tightness of the urethra and to resolve the issue of prolonged urethral drainage.

Upon reaching three months after the surgery, all patients at least once every six months or once a year had to undergo the standard assessment of the state established by the study protocol as follows: consultation with a urologist, clinical blood and urine tests, urethrography, urethroscopy, uroflowmetry, and ultrasound of the urinary system. Patients assessed the subjective status using the IPSS, QoL, IIEF-5 scales, and gave complaints.

There were several criteria to assess the treatment effectiveness: the maximum urine flow rate (by uroflowmetry), the diameter of the urethral lumen in the plastic zone (according to urethrography), the volume of residual urine (ultrasound assessment), and the indices of rating scales (IPSS, QoL, and IIEF-5). Successful (free from a relapse) were considered the results of treatment with the following parameters 3 months or more after surgery: *Q*_max_ more than 12 ml/sec; absence of residual urine, signs of recurrence according to urethrography data (normal diameter of the urethral lumen in the plastic zone is 5 mm or more); and absence of severe LUTS, unsatisfactory quality of life.

The treatment results were evaluated based on a comprehensive analysis of the parameters of all primary and secondary endpoints. The absence of relapse and adequate urination do not provide an accurate indication of satisfaction with treatment and the patient's quality of life after surgery. Therefore, interpretation of the results requires consideration of all factors.  Table 3 presents the treatment results divided into three groups.

### 2.5. Statistical Analysis

The initial data and surgical treatment results were analyzed using STATISTICA software for Windows version 10.0 (Statsoft, Inc, USA), SPSS Statistics version 23.0 (IBM, USA), and Stata version 16.0 (StataCorp, USA).

Simple and multiple logistic regressions helped to identify predictor variables for a binary response variable. To determine the predictors of post-operative complications development there was a univariate and multivariate logistic regression analysis. The predictor variables were selected according to the initial and closest parameters (partially presented in Tables 1, 2, and 4, more than 100 in total). Cox proportional risks regression helped to assess the correlation between one or more continuous or categorical variables and the time to an adverse event. The significance level for all methods is *p* ≤ 0.05.

### 2.6. Treatment Protocols

During the study, there were the following two different treatment protocols: standard (group I), when the patient was prohibited from drinking and eating on the day of surgery, underwent bowel cleansing the night before and the morning of surgery, and received a sedative (diazepam). Intraoperatively, absorbable suture material was used for individual interrupted sutures, including monopolar diathermocoagulation, separately interrupted skin suture, and standard dressings. On the first day after the surgery, only liquid intake was allowed, food intake was allowed from the second postoperative day. In the postoperative period, on the first day after the operation, anesthesia with narcotic analgesics was performed as needed. The patient was mobilized on the second day after the surgery. Infusion therapy was performed within the first 24–48 hours. Antibiotic therapy was carried out during the entire hospitalization. The minimum recommended hospital stay after surgical treatment was 7 days. The urethral catheter was removed 10–21 days after surgery.

Table 5 shows the FTS protocol scheme (group II).

The final choice of the method of surgical treatment was made in advance and corrected intraoperatively. The main types of operations were anastomotic urethroplasty (EPA) in the form of the classic Turner-Warwick/Webster operation [17, 18], vascular-sparing methods [19], intraurethral anastomosis [20], and buccal mucosa graft urethroplasty using various techniques (Asopa [21], Barbagli [22] and others), as well as the original minimally invasive technique [23].

All patients in both groups used neuraxial analgesia, prophylaxis of thromboembolic complications (low molecular weight heparins), and protection from stress ulcers (proton pump blockers). The severity of the postoperative pain syndrome was possible to assess according to the visual analog scale of pain (VAS) on the first day after surgical treatment.

Platelet-rich plasma (PRP method) and fibrin glue (i-PRF and Superfibrin method) were obtained by centrifugation (“Armed” centrifuge) in special tubes from the patient's peripheral venous blood.

### 2.7. A Clinical Example of the ERAS Protocol in Urethroplasty

Patient K., 62 years old, presented with the following complaints: inability to urinate adequately and presence of cystostomy. From the medical history it is known that urethral stricture was detected accidently. A cystostomy was inserted in 2021 due to acute urinary retention. TRUS revealed prostate volume of 20.7 cm^3^. An urethrogram on the 22.12.20 revealed a bulbo-membranous stricture of the urethra with narrowing of the lumen up to 1 mm. On December 29, 2020, a prostatic TUR and an internal optical urethrotomy were performed and the cystostomy was preserved. In the postoperative period urination was not restored. On control urethrocystoscopy and MSCT-urethrocystography the diagnosis was bulbo-membranous urethral stricture, recurrence, complication of underlying secondary chronic cystitis, chronic urinary retention, cystostoma, and hospitalized as planned for surgical treatment of urethral stricture.

Perioperative treatment was carried out according to the claimed method. Mini-invasive intraurethral urethroplasty with the use of oral mucosa graft was carried out (Vorobiev V.A., Beloborodov V.A. method of surgical treatment of urethral narrowing//patent No 2694477 from 15.07.19). Rehabilitation according to the claimed method was carried out.

In the preoperative period the detailed consultation about the possible principles of urethral strictures treatment, the reasons of their occurrence and consequences of refusal from treatment has been carried out. Possible alternative treatments such as endoscopic dissection, dilatation, anastomotic urethroplasty, and others are presented. An overview of the preoperative period, intraoperative nuances and a description of the expected state in the postoperative period and possible complications, as well as rehabilitation measures, are presented.

Immediately after the initial consultation, the patient's consent to surgical treatment according to the principles of accelerated recovery is obtained. An examination plan was prescribed as part of the accelerated pathway. All examinations were carried out on the following day within three hours. The indications and contraindications for surgery are reassessed on the basis of the examination results. A multidisciplinary team discussion was carried out as follows: the urologist, anesthetist, internist, radiologist, nurse, and rehabilitator. The possibility of adhering to the protocol on religious, ethical, social, and other grounds is assessed. Evaluated the need for prehabilitation: not identified. Recommended slagfree diet 2-3 days before surgery.

Patient is scheduled for surgery date. Hospitalization on the day of surgery, three hours before the planned surgical intervention. Self-preparation at home. Cleansing of bowel has not been carried out. Shaving of the operative field perineum, after pretreatment with skin antiseptic. Prevention of venous thromboembolism—compression knitwear and subcutaneous injection of Fraxiparin 0.3 ml.

On admission the patient was premedicated with celecoxib 100 mg, gabapentin 600 mg, and omeprazole 20 mg once oral. Carbohydrate loading of 200 ml of maltodextrose mixture orally was performed.

Antibacterial prophylaxis was carried out once 60 minutes before surgery according to the recommendations. Preoperative urine culture with pathogenic microflora growth and postoperative antibiotic therapy plan were made according to culture results.

Intraoperative method of anesthesia was epidural anesthesia. The operation time was 50 minutes. A 14Fr silicone urinary catheter was inserted. Intraoperative heating of the patient was carried out using an electric heating mattress. Heating of infusion solutions was carried out using a flow heater. Minimally invasive was perineal linear surgical access of 2 cm. The operation was performed using a 4.5x magnifying lens. Monopolar coagulation was not used. After performing a bulbar urethral access, a 2 cm transbulbar access was performed. After removal of scar tissues, a 3 × 1 cm oral graft was taken from the left cheek under local anesthesia with lidocaine 2% 20 ml solution. The defect was sutured with Vicryl 3–0. The graft was fixed in the formed bed with 5–0 monocryl continuous suture. The urethrotomy access is sutured with Monocryl 4–0. Sealing of the suture using Sulfacrylate adhesive was carried out. Reliable hemostasis must be achieved. No drainage was performed. Closure of the perineal access with Monocryl 4−0 in layers was carried out in continuous sutures. Cosmetic skin sutures was carried out with Sulfacrylate adhesive dressing. Intraoperatively, prevention of postoperative nausea and vomiting was performed—dexamethasone 4 mg and ondansetron 4 mg intravenously.

After the operation, the patient was transferred to a postoperative ward for 3 hours. Intraoperative pain management continued in the postoperative period according to the “no pain” principle, i.e. prevention rather than elimination of pain. After the end of the epidural anesthesia the patient was prescribed oral celecoxib 100 mg and acetaminophen 250 mg every 6–8 hours during the first postoperative day. The use of maltodextrose mixture is suggested one hour after surgery. Postoperative consumption of solid food is allowed 2 hours after surgery. Plant-based chewing gum is recommended to reduce the risk of postoperative functional bowel disorders and for the purpose of local antiseptic action.

The patient is activated 6 hours after the operation, following the cessation of epidural anesthesia. Activation involves sitting and walking. Glycemic control on the first and second post-operative days, no correction was necessary. On the second postoperative day, a control clinical blood count was performed. Postoperative antibiotic therapy was carried out according to the culture data—levofloxacin 500 mg once a day, 5 days. Wound treatment was not carried out. Independent daily hygiene-showering was recommended.

Prophylaxis of venous thromboembolism was continued—compression knitwear was worn until 21 days after the operation, plenty of fluids were administered and Fraxiparin 0.3 ml was injected intravenously until discharge. The patient was discharged from the hospital to outpatient care on the third postoperative day.

The urethral catheter was removed 7 days after surgery, after a control pericatheter urethrocystography to assess possible urine leakage from the access line.

Daily contact with the attending physician via phone calls and messenger for the first 10 days, then once every 2-3 days for up to a month. Monthly thereafter for one year. Check-ups and follow-ups ultrasound on the 3rd, 7th, 10th, 20th, and 30th day and then after 3, 6, and 12 months.

In 14 days after surgery, the use of the drug Longidase was recommended under the scheme of 1 suppository rectally once every 2 days, ^#^20.

No intraoperative or postoperative complications ≥ II according to Clavien-Dindo classification were registered in the patient. The patient was discharged in a satisfactory condition on the 3rd day after surgery.

According to the control urethrocystography and urethroscopy one year after surgery, there were no signs of urethral stricture recurrence. The diameter of the urethra in the urethroplasty zone was 6.5 mm. Quality of life indicators were in line with population averages.

### 2.8. Research Limitations

Limitations of the research were as follows: relatively small sample size, the average post-operative follow-up period of fewer than two years, single-center study, mixing of various surgical techniques, and different localizations of strictures within the protocol (anastomotic, substitution, and others).

## 3. Results

### 3.1. Immediate Results

To compare the immediate results of surgical treatment of urethral stricture disease after the standard treatment protocol (group I) and after the FTS protocol (group II) there was a comparative analysis of the postoperative parameters of the patients' condition and the examination results.

In the early and late postoperative periods, there were no cases of mortality in the two groups. In the early and late postoperative periods, there were no complications of anesthetic treatment or deterioration of the general somatic status. The need for mechanical ventilation or respiratory support did not arise in any case in the comparison groups. There were also no cases of heart failure that required inotropic support.

Intergroup analysis of the size of the surgical approach showed that its average linear dimensions of group I were 7.1 ± 2.1 cm, which is significantly greater than of group II (3.3 ± 1.2 cm, *p* < 0.001).

There was a chronometric analysis of the operating period. The average duration of surgery in groups I and II were 1.2 ± 0.35 hours and 1.1 ± 0.31 hours, respectively (*p*=0.273).

Table 4 shows the comparative characteristics of the postoperative state indicators of patients in the comparison groups.

The results analysis showed that in both comparison groups and postoperative complications in the early and late periods developed extremely rarely.

It should be noted that the development of incontinence and failure of the urethral suture occurred only in one case. Thus, univariate logistic regression analysis of these indicators did not reveal a statistically significant correlation with the initial parameters (*p*> 0.05) in the comparison groups.

Table 6 presents the information on predictive factors for the occurrence of post-operative complications (univariate and multivariate logistic regression analysis, the table includes signs with a level of *χ*^2^ > 1 and *p* < 0.05).

A simple (univariate) logistic regression analysis among 54 patients of both comparison groups revealed that some indicators acquired special significance in predicting the development of subfebrile condition in the early post-operative period – the results are presented in Table 6.

The obtained results helped to build a model for predicting subfebrile status in multivariate regression analysis (selection from predictor factors with a significance level of *p* < 0.05). Low subjective assessment of the quality of life (more than 4 points; Coefficient 1.5; 95% CI 0.1; 2.9; *p*=0.035), as well as the size of the surgical access (for each 1 cm; Coefficient 1, 09; 95% CI 0.2; 1.98; *p*=0.016). The rest of the factors were not significant (*p* > 0.05).

The independent predictors of persistent pain syndrome found by simple logistic regression analysis (presented in Table 6) were included in further analysis. Localization of the urethral stricture in the penile part of the urethra (Coefficient 5.41; 95% CI 0.55; 10.2; *p*=0.029), as well as the size of the surgical access (for each 1 cm; Coefficient 1.29; 95% CI 0.45; 2.13; *p*=0.002), became significant predictors of persistent pain syndrome. The rest of the factors were not significant (*p* > 0.05).

Table 7 shows postoperative indicators of urodynamics, objective, and functional status (IPSS, QoL, and IIEF-5) in the comparison groups.

There was a comparative intergroup analysis of these indicators. The groups were comparable according to the results of an objective examination, the state of urodynamics, and functional status in the early and late postoperative periods (*p* > 0.05).

### 3.2. Long-Term Results

Long-term results of surgical treatment of stricture disease were possible to assess according to the control endpoints of the examination. The average period of clinical observations was 468 days with 95% CI 423–513, and the maximum period was 721 days.  Table 8 presents general indicators of the long-term postoperative period in the comparison groups.

For group I, the average observation period was 469 days with a 95% CI of 401–537 days (maximum period of 705 days). For group II, the average observation period was 466 days with a 95% CI of 403–529 days (maximum period of 721 days).

In the long-term postoperative period, there was a single episode of mortality in group I, which was not associated with surgery.

There were few significant complications in the long-term postoperative period. In 3 (10.3%) cases from group I and 2 cases (8%) from group II, there was an acute inflammatory process of the genitourinary system in the period from 3 to 12 months, which required the antibacterial drugs (*p*=0.786).

7 (24.1%) patients of group I and 5 (20%) patients of group II (*p*=0.770) presented complaints of penile shortening during active questioning 3 months after surgery.

To determine the predictors of the development of the above complications, there was univariate and multivariate logistic regression analysis. The predictor variables were selected according to the initial and closest parameters (partially presented in Tables 1, 2, 5, and 7, more than 120 in total). Information on predictive factors for the occurrence of postoperative complications in the long-term postoperative period (univariate and multivariate logistic regression analysis, the table includes signs with *χ*^2^ > 1 and *p* < 0.05) are presented in Table 9.

The obtained results allowed constructing a model for the prognosis of infectious complications in the long-term postoperative period in multivariate regression analysis (selection from factors *p* < 0.05). Decompensation of diabetes mellitus in the long-term postoperative period (Coefficient 3.92; 95% CI 0.09; 7.7; *p*=0.045) was a significant predictor of this complication. Consequently, after performing urethroplasty surgeries, the risk of developing infectious complications in the long-term period increases with decompensation of diabetes mellitus.

The obtained results helped to construct a model for predicting penile shortening in multivariate regression analysis (selection from factors *p* < 0.05). A significant predictor of this complication was the length of the urethral stricture more than 3 cm (coefficient 2.87; 95% CI 0.08; 5.67; *p*=0.043). Consequently, urethroplasty for patients with a urethral stricture longer than 3 cm increases the risk of penile shortening.

**Figure 2:**
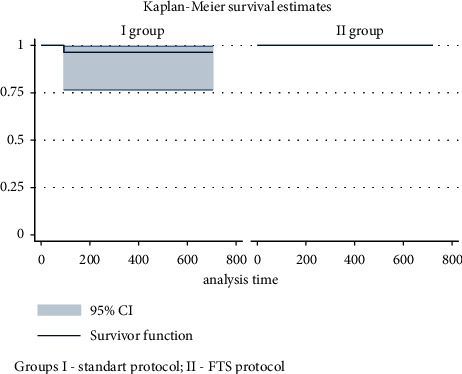
Kaplan–Meier curve (the ratio of the number of patients without death to the duration of follow-up) of patient survival in the comparison groups.

There was a statistical analysis of overall survival. The Kaplan–Meier estimates of the survival rate of group II patients during the entire observation period after urethroplasty were equal to 100%. The values of the Kaplan–Meier estimates of the survival rate of group I patients during the entire observation period (up to 2 years) were 96.3 ± 3.63% with a 95% CI of 76.4–99.4%. The log-rank test did not reveal statistically significant differences in the survival rate in the long-term post-operative period in the comparison groups (*p*=0.335), which is graphically presented by the Kaplan–Meier method in Figure 2.

Cox proportional hazards regression analysis showed no reliable predictor of mortality in the long-term post-operative period. Probably, such a result of the statistical analysis is due to the small representation of the factor (a single case of mortality).

The analysis of the dependence of mortality on general surgical technical reasons was not performed due to the absence of such. The survival rate for this parameter for both groups was 100% over the entire observation period.

Table 10 presents comparative information on the success of surgical treatment in the comparison groups.

In the first group, out of 29 primary operations, 25 were successful (86.2%). In the second group, 23 out of 25 primary operations were successful. Thus, the primary efficiency was 92%.

The Kaplan–Meier estimates the absence of recurrence of urethral stricture in group I as 93.1 ± 4.7% during the first three months (95% CI 75.1–98.2%), after a year–89.5 ± 5, 7% (95% CI 70.9–96.5%) and after two years—78.3 ± 11.6% (95% CI 44.8–92.8%). These indicators for group II during the first three months were 100% and after one and two years —90.3 ± 6.5% (95% CI 66.3–97.5%).

**Figure 3:**
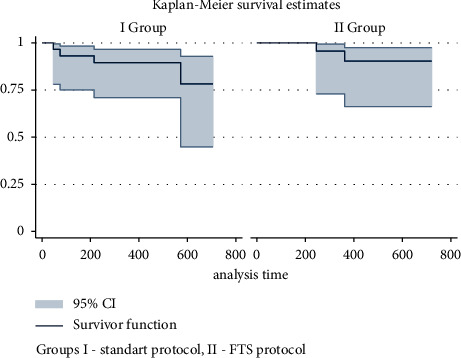
Absence of relapse in study groups according to the Kaplan–Meier method.

The log-rank test did not reveal statistically significant differences (*p*=0.512; *χ*^2^ = 0.43) in the recurrence rate over the entire observation period, which is graphically expressed by the Kaplan–Meier method in Figure 3.

The predictor variables were selected according to the initial parameters, as well as according to the control parameters in the postoperative period.  Table 11 presents a Cox proportional hazards regression model showing the effect of variables on recurrence risk.

Multivariate Cox proportional hazards regression analysis (sample of *p* < 0.05) demonstrated the significance of urethral suture failure (HR 4.36; 95% CI 1.6; 7.11; *p*=0.002) in predicting a possible recurrence after urethroplasty surgery.

Analyzing the comparative intergroup effectiveness of the treatment based on the criteria established in the study, good treatment results obtained 5 (17.2%) patients of group I and 20 (80%) patients in group II (*p*=0.0049). Satisfactory results obtained 20 (68.9%) patients of group I and 3 (12%) patients of group II (*p*=0.0055). Unsatisfactory results obtained 4 (13.7%) patients in group I and 2 (8%) patients in group II (*p*=0.544).

Therefore, the use of the FTS protocol after urethroplasty allows achieving better treatment results than the classical approach. Both methods of urethroplasty had zero operating and hospital mortality and a low incidence of postoperative general and urinary system-related complications. Both methods are highly effective and safe. However, the classical approach is associated with longer disability and worse subjective perception and objective assessment of the perioperative period for patients.

## 4. Discussion

Modern surgery, including urology, has many tools and techniques that facilitate the perioperative period for both the patient and the healthcare organization. However, the complexity of preoperative diagnosis, its duration and cost, and operational and postoperative surgical stress reduce the quality of medical care. Urology is one of the leaders in minimal invasiveness, making the most of endovideosurgery and endoscopy. That is why, according to the authors, of particular interest is the development of protocols for enhanced recovery in urology. Thus, a typical urological problem postoperative pain remains relevant. The rehabilitation and restoration of working capacity also remain relevant.

A few interesting results were obtained in the presented study. The overall efficacy of urethroplasty operations, regardless of the treatment protocol, was comparable (*p*=0.870), as well as the likelihood of recurrence within 2 years (*p*=0.512). The results obtained are consistent with the data of other authors; the average efficiency of urethroplasty operations, regardless of the location and length of the stricture, when using anastomotic or replacement methods is 85 ± 10% [24–27].

In the development of relapse, a special role (according to multivariate Cox proportional hazards regression) played technical complications and failure of the urethral suture (OR 4.36; 95% CI 1.6; 7.11; *p*=0.002). The FTS protocol for urethroplasty surgery offers several simple techniques to minimize this risk. First, it is proposed to use only a continuous and sealed urethral suture; second, the suture is additionally treated with fibrin glue; and third, the surrounding tissues are infiltrated by platelet-rich plasma. The use of platelet-rich plasma can reduce the zones of necrosis, the severity of local inflammation, and improve angiogenesis, which has been confirmed by several works [16, 28, 29]. Fibrin glue provides better tightness, reliable fixation of the graft, reduces the risk of periurethral leakage [14], and the risk of fistula formation [30]. There is evidence of the successful use of a mixture of fibrin and cyanoacrylic glue for the treatment of vesicourethral anastomoses leak after prostatectomy [31].

Predictors of complications were assessed separately. Thus, the use of anastomotic methods of urethroplasty increases the risk of complaints of shortening of the penis with strictures longer than 3 cm. Decompensation of diabetes mellitus in the postoperative period increases the likelihood of an acute infection of the genitals and lower urinary tract. Without considering the data of univariate analysis, the results obtained are consistent with the results of similar studies [32, 33]. Separately, it should be noted the effect of the size of the surgical approach on the severity of postoperative pain. Such conclusions have been repeatedly confirmed by early works [34].

Logistic analysis of predictors was performed in order to establish a possible relationship between elements of the FTS protocol and treatment outcomes. The negative impact of pathological hyperglycemia and the use of narcotic analgesics on the likelihood of developing subfebrility confirm the expediency of including in the protocol such elements as minimizing the use of opiate analgesics and performing strict control of glycemia and its mandatory correction. In turn, minimizing surgical trauma directly affects the need for analgesics and the risks of other complications.

An excellent result is considered a significant decrease in the severity of post-operative pain (*p* < 0.001), and, consequently, a decrease in surgical stress, as well as a reduction in the treatment period, from the moment of diagnosis to full recovery (*p* < 0.001) when using the developed FTS protocol. The authors did not find similar studies of the effectiveness of FTS in urethroplasty; however, the general concept of the system and many works [35–41] demonstrate similar results.

The evaluation of treatment outcomes according to the most rigorous analysis of subjective perception and objective status (such as general perception of treatment, the severity of pain, and general indicators of success and risks of complications) shows that the FTS protocol provides superior results. Good results obtained 80% of Group II patients compared with 17.2% of Group I patients, *p*=0.0049. Thus, we consider it expedient to introduce the developed FTS protocol in specialized urethroplasty centers with the aim of a further in-depth multicenter study of its effectiveness.

Distinctive features of our study are prospective recruitment of patients, distribution into groups by randomization; homogeneity of groups in terms of initial characteristics, similar morphological characteristics of patients; mandatory diagnostic algorithm before surgery and post-operative control for all patients; description of the patient management algorithm with a detailed presentation of materials and research results.

## 5. Conclusion

Both treatment protocols are safe, effective, and bring minimal risks of complications. Both protocols lead to the restoration of independent adequate urination equally (86.2% vs 92%; *p*=0.870). The use of the “fast track surgery” protocol in urethroplasty with generally similar treatment results makes it possible to achieve a better functional and objective condition of patients in the postoperative period due to less pain, shorter catheterization, and hospitalization.

## Figures and Tables

**Table 1:** Comparative characteristics of patients in comparison groups before surgery.

Indicator	Group I (*n* = 29)	Group II (*n* = 25)	*p*
*General indicators*:			
Age (years)	51.0 (±16.8)	53.6 (±13.1)	0.527
Height (cm)	173.4 (±6.8)	172.8 (±5.0)	0.719
Weight (kg)	73.8 (±12.7)	81.8 (±17.4)	0.057

*Anamnesis*:			
Duration of the established disease (months)	24 (6; 60)	24 (12; 60)	0.645
Smoking, *n* (%)	18 (62.0%)	15 (60%)	0.939
Contacts with harmful substances, *n* (%)	6 (20.6%)	6 (24%)	0.816
Allergic anamnesis, *n* (%)	2 (6.8%)	3 (12%)	0.557
Cystostomy, *n* (%)	13 (44.8%)	9 (36%)	0.668

*Urological operations and manipulations*:			
Traumatic catheterization, *n* (%)	9 (31.0%)	6 (24%)	0.664
Bougie of the urethra, *n* (%)	10 (34.4%)	10 (40%)	0.776
Optical urethrotomy, *n* (%)	7 (24.1%)	7 (28%)	0.804
EPA, *n* (%)	10 (34.4%)	4 (16%)	0.232
BMG urethroplasty, *n* (%)	6 (20.6%)	3 (12%)	0.468
TUR of the prostate, *n* (%)	5 (17.2%)	2 (8%)	0.374
TUR of the bladder, *n* (%)	2 (6.8%)	4 (16%)	0.343
Prostate surgery, *n* (%)	0 (0%)	3 (12%)	0.070

*The etiology of urethral stricture*:			
Trauma, *n* (%)	12 (41.3%)	6 (24%)	0.336
Iatrogenic, *n* (%)	14 (48.2%)	14 (56%)	0.750
The inflammatory, *n* (%)	1 (3.4%)	2 (8%)	0.491
Idiopathic, *n* (%)	2 (6.8%)	3 (12%)	0.557

*Concomitant diseases*:			
Ischemic heart disease, *n* (%)	7 (24.1%)	10 (40%)	0.367
Hypertension, *n* (%)	10 (34.4%)	12 (48%)	0.514
Diabetes mellitus, *n* (%)	2 (6.8%)	4 (16%)	0.343
Chronic urinary infection, *n* (%)	11 (37.9%)	4 (16%)	0.173
Hyperplasia of the prostate, *n* (%)	9 (31.0%)	6 (24%)	0.664

*Medications*:			
Medicines affecting erection, *n* (%)	7 (25%)	9 (36%)	0.484
A-blockers, *n* (%)	5 (17.8%)	3 (12%)	0.640

Note: EPA-excision and primary anastomosis; BMG-buccal mucosa graft; TUR-transurethral resection.

**Table 2:** The results of an objective examination and the state of the functional status.

Indicator	Group I (*n* = 29)	Group II (*n* = 25)	*p*
*Localization of the structure*:			
Glans urethra, *n* (%)	2 (6.8%)	3 (12%)	0.557
The penile urethra, *n* (%)	6 (20.6%)	1 (4%)	0.107
The bulbar urethra, *n* (%)	18 (62.0%)	18 (72%)	0.730
The membranous urethra, *n* (%)	13 (44.8%)	12 (48%)	0.887
Length of stricture, (mm)	20 (10; 40)	15 (10; 25)	0.780
Minimum diameter of the urethra, (mm)	1 (1; 2)	1 (1; 2)	0.277
*Q *_max_, (ml/s)	7.1 (±2.6)	7.2 (±2.2)	0.920
Volume of residual urine, (ml)	150 (90; 240)	130 (45; 230)	0.564
Fistulas or diverticula of the urethra, *n* (%)	3 (10.3%)	1 (4%)	0.408
Prostate volume, (cm3)	23.3 (±8.8)	26.0 (±7.9)	0.262
IIEF-5, score	14 (9; 18)	12 (8; 16)	0.423
Qol, score	5 (4; 5)	5 (4; 6)	0.167
IPSS, score	26 (23; 30)	28 (26; 34)	0.095

Note: *Q*max-maximum flow rate; IIEF-Iinternational Index of Erectile Function; QoL–the quality of life; IPSS-International Prostate Symptom Score. Thus, the analysis of the main characteristics of patients in the comparison groups demonstrated their comparability (*p* > 0.05).

**Table 3:** Evaluation of treatment results using the combined (multiple) endpoint methods.

Parameter	Good	Satisfactory	Unsatisfactory
Absence of relapse	+	+	−
No severe pain syndrome (>5 points on the pain scale) in the post-operative period	+	−	−
No complications	+	−	−
Satisfaction with treatment	+	−	−

**Table 4:** Comparative data of the nearest results in patients of the comparison groups.

Indicator	Group I (*n* = 29)	Group II (*n* = 25)	*p*
Hematoma in the operation area, *n* (%)	0	0	0
Graft necrosis, *n* (%)	0	0	0
Urethrorrhagia, *n* (%)	0	0	0
Subfebrile condition in the early postoperative period, *n* (%)	27 (93.1%)	7 (28%)	0.014
Infectious complications, *n* (%)	0 (0)	0 (0)	0
Urethral suture failure, *n* (%)	1 (3.4%)	0 (0)	0.356
Constant pain syndrome (VAS more than 5 points) on the first day after surgery, *n* (%)	23 (79.3%)	1 (4%)	0.003
Postoperative pain level on the first day, points	8 (6; 9)	4 (4; 5)	<0.001
The need for narcotic pain relief, *n* (%)	13 (44.8)	1 (4%)	0.007
Average length of hospital stay, bed-day	11.5 ± 3.4	3.8 ± 1.2	<0.001
Terms of catheterization, days	15.1 ± 3.3	6.2 ± 1.8	<0.001
Total time spent on treatment, days	27.3 ± 5.2	13.7 ± 2.4	<0.001
Postoperative incontinence due to surgery, *n* (%)	1 (3.4%)	0	0.356
Continent after surgery, *n* (%)	28 (96.5%)	23 (92%)	0.902
Satisfaction with the performed treatment, *n* (%)	17 (58.6%)	21 (84%)	0.397

**Table 5:** The FTS protocol for the supervision of patients with urethroplasty for urethral stricture.

FTS protocol for the urethroplasty:		
Preoperative period	Intraoperative period	Postoperative period
Informing the patient about the disease, treatment options, and possible outcomes, indicating the average effectiveness, risks of complications, typical post-operative condition, timing of catheterization, hospitalization, possible methods of prerehabilitation, and further rehabilitation methods	Preferred method of anesthesia-local anesthesia/multimodal anesthesia	Early fluids intake (2-3 hours after surgery) and food (6 hours after surgery)

One-day concept-the patient undergoes most of the preoperative examinations in one day, without the need for multiple repreparation. The order of examinations and tests is optimized and sorted to achieve the desired outcome		

Rigorous evaluation of indications for surgical treatment: *Q*_max_ < 12 ml/s Urethral lumen diameter <4 mm Presence of residual urine Presence of urethral distraction defect	Heating of the patient during the operation with the control of normothermia	Early activation (6–8 hours after surgery, after evaluation by an anesthesiologist)

Assessment of the possibility of patient compliance with the protocol and its feasibility in the medical institution	Heating of infusion solutions and inhalation gases	Physical therapy (breathing exercises, walking, and other exercises)

Preventive administration of antihistamines and antacids drugs	Minimally invasive surgical approaches	Multimodal prevention of nausea and vomiting (metoclopramide + ondansetron)

Refusal of preoperative sedation	The rejection of the use of monopolar coagulation and resection	Removal of the urethral catheter after performing pericatheter urethrography no later than the seventh day after surgery

Prerehabilitation based on indications: Age group Obesity Exhaustion The sarcopenia Impaired carbohydrate tolerance or diabetes mellitus	Application of bipolar coagulation	Use of drugs that improve microcirculation, reparants, and hyperbaric oxygenation (in the mode of 1.0–1.5 ATM, for 45 minutes, 5–10 sessions; in the absence of contraindications)

Preoperative antibiotic therapy according to the testimony: the presence of latent or obvious infection of the genitourinary system (according to the results of bacteriological research, real-time PCR, and infection of other organs	The rejection of coagulation on the spongy body of the urethra	The use of enzyme drugs (longidase, rectal suppositories) after 14 days after surgery in courses of 20 pieces with an interval of 2 days every 6 months

Multidisciplinary examination of patients: Urologist Anesthetist ENT doctor Dentist General practitioner/Cardiologist The radiologist physical therapy doctor And other specialists as needed	Sealed continuous urethral suture with 4–6/0 monofilament thread	Continuation of prevention of thromboembolic complications by compression of the lower extremities and the use of low-molecular-weight heparins

Performing CT/MRI of the small pelvis, CT/RI MRI of the urinary system, CT/RI MRI-urethrography with 3D-modeling, assessment of the state of the bone and joint apparatus of the pelvis, organs	Use of platelet-rich plasma as injections into the submucosal layer of the urethra and surrounding tissues	Multimodal analgesia for pain control (Dexketoprofen + paracetomol)

A rich carbohydrate diet (if there are no contraindications) and 200 ml of liquid protein 2.5 hours before surgery	Use fibrin glue locally at the urethral seam	Use of chewing gum on the first and second day after surgery

The last meal during the operation in the morning hours at 21-22 hours the day before, during the operation in the afternoon no later than 6 hours before the operation	Use of silicone urethral catheters 14–16 ch	Monitoring of blood and urine parameters on the first day after surgery

Antibiotic prophylaxis 60 minutes before surgery with 3rd generation cephalosporins	The rejection of the use of drains	Strict glycemic control in patients with impaired carbohydrate tolerance and diabetes mellitus

Shaving of the surgical field with subsequent treatment with solutions of skin antiseptics	Sealed cosmetic skin seam with no loose ends or knots on the skin	A detailed discussion of the behavior of the patient and the rehabilitation plan before the discharge

Oral rinsing with an aqueous solution of chlorhexidine during planned urethroplasty using buccal mucosa graft	The adhesive bandage on the skin	Detailed written instructions in the discharge documents

Prevention of thromboembolic complications by compression of the lower extremities and administration of low-molecular-weight heparins	Intraoperative euvolemia	Strict plan of follow-up examinations in the post-operative period

Avoiding the use of cleansing enemas		Strict adherence to postoperative hygiene of the genitals (when using an adhesive bandage, the patient is recommended to take a hygienic shower daily from the second day)

Transfer of the patient to a slageless diet 2-3 days before surgery		Discharge from the hospital 1–3 days after the operation with the transfer of the patient to outpatient observation

Preparation of the intestines with laxatives or once microclysters		Recommended return to work 2 days after removal of urethral catheter

**Table 6:** Predictors of postoperative complications.

Complications in the early and late post-operative period	Predictor	Univariate analysis			Multivariate analysis	
		*χ * ^2^	Coefficient (95% CI)	*p*	Coefficient (95% CI)	*p*
Subfebrile Condition Multivariate logit regression: *χ*^2^ = 45.75; *p* < 0.0001	Standard protocol	26.99	3.5 (1.8; 5.2)	<0.001	2.3 (−1.2; 5.9)	0.208
	The quality of life	5.54	0.76 (0.06; 1.4)	0.033	1.5 (0.1; 2.9)	0.035
	Diabetes	6.16	2.3 (0.16; 4.6)	0.035	3.4 (−3.6; 10.4)	0.340
	Vascular atherosclerosis	4.97	1.3 (0.14; 2.5)	0.029	1.3 (0.94; 3.6)	0.246
	Surgical access size	28.07	0.85 (0.39; 1.3)	<0.001	1.09 (0.2; 1.98)	0.016
	Pain more than 5 points on the VAS scale	17.04	2.8 (1.1; 4.4)	0.001	0,77 (−10,4; 3,6)	0.874
	Use of narcotic analgesics	8.63	2.4 (0.33; 4.5)	0.023	3.3 (−6.6; 13.4)	0.509

Persistent pain syndrome, >5 points on the VAS scale Multivariate logit regression *χ*^2^ = 7,78; *p* < 0.001 (0.0001)	Pelvic bone injury (history)	10.88	2.8 (0.66; 4.9)	0.010	2.6 (−2.0; 7.35)	0.263
	Progression of urethral stricture	8.61	1.77 (0.5; 3.02)	0.005	0.3 (−2.6; 3.2)	0.841
	Urohematomas (history)	7.5	1.8 (0.41; 3.3)	0.011	0.99 (−1.9; 3.9)	0.514
	Penile strictures	5.89	2.2 (0.7; 4.46)	0.043	5.41 (0.55; 10.2)	0.029
	Duration of surgery	5.88	2.1 (0.27; 3.9)	0.024	2.3 (−7.4; 2.7)	0.364
	Surgical access dimensions	44.14	1.2 (0.6; 1.77)	<0.001	1.29 (0.45; 2.13)	0.002

Note: VAS-a visual analog scale of pain.

**Table 7:** Postoperative indicators of urodynamics, objective, and functional status (IPSS, QoL, and IIEF-5) in comparison groups.

Parameter	Group I (*n* = 29)	Group II (*n* = 25)	*p*
IPSS, score	5 (2; 8)	4 (2; 7)	0.564
IIEF-5, score	15 (11; 18)	14 (9; 18)	0.794
QoL, score	1 (1; 2)	1 (1; 1)	0.187
*Q *_max_, (ml/sec)	15.5 ± 2.7	17.0 ± 3.1	0.065
Residual urine volume, ml	0 (0; 0)	0 (0; 0)	0.931
Achieved urethral lumen diameter in the operation area, mm	5.1 ± 1.2	5.6 ± 1.3	0.106

Note: IPSS-International Lower Urinary Tract Symptom Scale; IIEF-5-International Index of Erectile Function; QoL-the quality of life; *Q*_max_-maximum urine flow rate.

**Table 8:** General indicators in the long-term period.

Indicator	Group I (*n* = 47)	Group II (*n* = 47)
Dropout patients, *n* (%)	18 (38.2%)	22 (46.8%)
The number of patients who completed the study, *n* (%)	29 (61.7%)	25 (53.1%)
Nonlethal patients, *n* (%)	28 (96.5%)	25 (100%)
Long-term general mortality, *n* (%)	1 (3.4%)	0 (0%)
Urological mortality, *n* (%)	0 (0)	0 (0)
Relative risk of death, (%)	I/II–98.182%	
Reducing absolute risk	I/II–1.818%	
Reducing relative risk	I/II–54.545%	
A chance to die in a distant period	3.4%	0%

**Table 9:** Predictors of the complications in the long-term postoperative period.

Complications in the long-term postoperative period	Predictor	Univariate analysis			Multivariate analysis	
		*χ * ^2^	Coefficient (95% CI)	*χ * ^2^	Coefficient (95% CI)	*χ * ^2^
Long-term infectious complications multivariate logit regression *χ*^2^ = 16.58; *p*=0.0110	Bladder cancer (history) diabetes mellitus, and decompensation CVI Vascular atherosclerosis Urethral lumen diameter (final) Qmax (final)	4.02 12.59 5.08 5.57 4.12 5.55	2.3 (0.18; 4.46) 3.8 (1.3; 6.22) 2.75 (0.47; 5.03) 2.4 (0.12; 4.68) −0.6 (−1.3; −0.08) −0.3 (−0.5; −0.04)	0.033 0.002 0.018 0.039 0.047 0.020	−1.6 (−7.1; 3.7) 3.92 (0.09; 7.7) 2.2 (−2.4; 6.9) 0.07 (−3.7; 3.8) −0.27 (−2.6; 2.1) −0.08 (−1.0; 0.8)	0.543 0.045 0.343 0.969 0.826 0.866

Shortening of the penis multivariate logit regression *χ*^2^ = 29.28; *p*=0.0003	Post-traumatic stricture Pelvic injury Distraction defect of the urethra Cystostomy Previous EPA Unsuccessful treatment (history) Inadequate EPA (history) Urethral stricture > 30 mm	11.60 13.57 19.55 7.53 4.27 7.36 8.86 15.51	2.39 (0.89; 3.8) 2.87 (1.2; 4.51) 4.05 (1.75; 6.3) 1.9 (0.43; 3.3) 1.4 (0.07; 2.81) 1.85 (0.46; 3.25) 2.12 (0.69; 3.56) 2.94 (1.36; 4.52)	0.002 0.001 0.001 0.011 0.038 0.009 0.004 <0.001	2.03 (−1.0; 5.1) 1.78 (−2.1; 5.7) 0.06 (−4.4; 4.5) 1.4 (−1.03; 3.99) −0.79 (−4.5; 2.9) 0.11 (−2.8; 3.03) 0.26 (−3.3; 3.87) 2.87 (0.08; 5.67)	0.202 0.377 0.977 0.249 0.682 0.939 0.884 0.043

Note: CVI-chronic venous insufficiency; *Q*_max_-maximum flow rate; EPA-anastomotic urethroplasty.

**Table 10:** Comparative data on the success of urethroplasty operations in the comparison groups in the long-term period.

Indicator	Group I (*n* = 29)	Group II (*n* = 25)	*p*
Successful primary, *n* (%)	25 (86.2%)	23 (92%)	0.870
True relapse, *n* (%)	4 (13.7%)	2 (8%)	0.544

**Table 11:** Cox proportional hazards regression model.

Variable	Univariate Cox analysis			Cox multivariate analysis, *χ*^2^ = 13.19; *p*=0.0217	
	Wald *χ*^2^	HR (95% CI)	*p*	Or (95% CI)	*p*
Hemorrhoids	5.80	8.4 (1.6; 41.8)	0.009	2.35 (−0.05; 4.77)	0.055
Bladder cancer (history)	3.19	6.1 (1.02; 36.87)	0.046	1.14 (−2.1; 4.43)	0.493
Chronic venous insufficiency	3.3	6.1 (1.12; 33.5)	0.036	−0.93 (−4.6; 2.8)	0.625
Inconsistency of the urethral suture	4.17	25.6 (2.32; 282.9)	0.008	4.36 (1.6; 7.11)	0.002
Infectious complications	3.07	5.64 (1.03; 30.92)	0.046	1.0 (−2.14; 4.15)	0.513

## Data Availability

The data used for supporting this study will be available from the corresponding authors upon request.
